# Has the pandemic made us more digitally literate? Innovative association rule mining study of the relationships between shifts in digital skills and cybersecurity awareness occurring whilst working remotely during the COVID-19 pandemic

**DOI:** 10.1007/s12652-022-04371-1

**Published:** 2022-08-11

**Authors:** Aleksandra Pawlicka, Renata Tomaszewska, Ewa Krause, Dagmara Jaroszewska-Choraś, Marek Pawlicki, Michał Choraś

**Affiliations:** 1 ITTI Sp. z o.o., Poznań, Poland; 2 Faculty of Pedagogy, Kazimierz Wielki University, Bydgoszcz, Poland; 3 Institute of Law and Economy, Kazimierz Wielki University, Bydgoszcz, Poland; 4 Bydgoszcz University of Science And Technology, Bydgoszcz, Poland

**Keywords:** Covid-19 pandemic, Cybersecurity, Digital skills, Lifelong learning

## Abstract

Digital literacy has been included in the set of the eight key competences, which are necessary to enjoy life to the full in the twenty-first century. According to the previous studies, women tend to possess lower digital competence than men; the older the person, the lower the level of digital literacy. To date, Polish citizens in general have worse skills than the European average. This may lead to people being socially excluded and vulnerable to cybersecurity threats, especially in the times of the COVID-19 pandemic, which requires them to work, study and shop using the Internet. The study concerned Polish women who work at universities, as scientists and teachers. Their perceived level of their digital literacy has been studied in the broad campaign, along with their awareness of the cybersecurity matters. Then, the collected results were processed with an association rules mining algorithm, uncovering the factors related to the shifts in them.

## Introduction

The development of the Internet and information technologies has changed people’s lives in innumerable ways. Citizens are able to work more efficiently, purchase goods from the comfort of their homes, access any kind of information and knowledge, communicate instantly with people all around the globe, and so on. Thanks to the technology, they are able to live healthier, longer, safer and happier lives. In today’s modern world, one needs to know how to utilize technologies to their advantage; it has become the requirement for the active participation in society. In other words, digital literacy has become a life skill, or a key competence (Telecentre Europe 2014).

The COVID-19 pandemic that we are yet to see the end of has transformed virtually every aspect of people’s lives. In some fields the shifts have been subtle, some others have been changed drastically and people may still be in the process of adapting to the new reality. One of the major changed consisted in transferring business, schooling and commerce to the on-line domain, to an unprecedented scale. Hardly anybody was prepared for this change; the results of it were difficult to predict, too. After a number of months passed, the time came for some initial reflections upon the consequences.

Thus, the multidisciplinary research team has decided to conduct a series of studies and scientifically check whether the pandemic’s side effect has indeed been the increase in the level of digital literacy (as suggested by the media, e.g., in Jeleński (2020)), and if the malicious activity of hackers during the crisis have made people be more aware of the dangers and threats that using the cyberspace may pose. Additionally, the factors influencing both aspects were to be uncovered. The rationale behind the selected topic, study group and methods will be presented in the further part of this paper.

The study included two major steps. Firstly, the team has conducted a broad campaign (the first of the kind) involving as many as 380 women working at Polish universities in order to construct a dataset. Then, an association rule mining experiment was conducted, revealing the actual relationships between the studied items, in order to answer the research questions stated in Sect. 2. The remainder of this paper is structured as follows: The remainder of Sect. 2 provides the rationale and context for the study, whilst Sect. 3—the theoretical background for the study. The materials, methods and the course of the study are presented in detail in Sect. 4. Then, the results of the study have been outlined, followed by the Discussion of the results and threats to validity in Sect. 5. Finally, the closing remarks are presented in Sect. refs6.

## The research questions, and the rationale and the context for the study

### Research questions

The study aimed at answering four research questions. They will be presented in this section, whilst the rationale and the motives will be explained thereafter.

#### Research question 1

Does the compulsory remote work make the perceived level of one’s digital competence increase? The assumed answer: yes, it does. In order to build up a more comprehensive picture of the matter, the research team wished to know if the workplace/employer of the respondent provided any kind of training or support for the workers doing remote job, and if its presence or lack thereof influences the perceived level of one’s digital literacy.

#### Research question 2

Does the compulsory remote work make the perceived level of one’s cybersecurity awareness increase? The assumed answer: yes, it does. Additionally, the team wished to see whether employers help remote workers gain knowledge of the cyberspace threats and the ways of preventing them.

#### Research question 3

Is the level of perceived digital literacy related to the age of the respondent? The assumed answer: yes, it does. Based on the quoted study by EUROSTAT, the younger the person, the higher their level of digital literacy. The study aimed at checking whether younger people would assess their skills in a significantly better way than the older ones, or if the oldest respondents would assess their skills lower than the younger ones.

#### Research question 4

Does a higher level of cybersecurity awareness relate to feeling safer when working online, or vice versa? Does training at one’s workplace contribute to the feeling of security?

The research questions have been summed up in Table 1.

**Table 1:** The main research questions of the study

No.	Research question
1	Does the compulsory remote work make the perceived level of one’s digital competence increase?
2	Does the compulsory remote work make the perceived level of one’s cyberse-curity awareness increase?
3	Is the level of perceived digital literacy related to the age of the respondent?
4	Does a higher level of cybersecurity awareness relate to feeling safer when working online, or vice versa?

### Current context for digital literacy of women in the Republic of Poland

Despite digital skills being deemed a necessity in the twenty-first century, according to the most recent study by Eurostat, merely 58% of Europeans aged 16–74 possess basic or above basic digital skills. There is the difference to be noticed between genders; 60% of males possess the skills, whilst the percentage of women is 4% lower. In Poland, while the overall percentage of the people having basic or above basic digital skills (44%, 22^nd^ place amongst EU28 countries) may not be drastically lower than the European average, there again exists the difference between genders, and only 43% women aged 16–74 are digitally literate.

In addition to this, it must be noted that another study has shown that the individuals possessing digital skills are mostly the very young ones, aged 16–19 (European average: 83%, Poland: 84%). Although the study has encompassed only the people aged up to 29 years old, it can be clearly visible that the older the individuals, the fewer of them possess the digital skills - for the group aged 20–24 it was 81% for Europe and 76% for Poland, whilst in the group aged 25–29, it was 78% and 69%, respectively (EUROSTAT, 2020). Taking the above-mentioned into consideration, it may thus be concluded that the group which may possess the lowest digital skills are the middle aged and elderly women. In the light of the fact that digital literacy is considered one of the life skills, the lack thereof may make one’s enjoying civic rights harder, or even lead to social exclusion (Soomro et al. 2020).

### Current context of the Covid-19 Pandemic

When WHO first declared the outbreak of the COVID-19 disease a Public Health Emergency of International Concern in January 2020, and then, subsequently a pandemic in March 2020, no person probably imagined how life was going to change in the upcoming weeks and months.

In the struggle to prevent the disease from spreading, many drastic measures have been taken, including lockdowns and compulsory social distancing. This meant that schools, businesses and countless other organizations had to start working remotely, utilizing cyberspace and digital tools. Millions of people were forced to turn to the online mode overnight, no matter if they had the skills to do it or not, and no matter if they even had a computer or access to the Internet (Pawlicka et al. 2021b). For many, this meant they suddenly lost the ability to perform their professional duties, or their access to education was denied. After several months have passed, and governments, companies and individuals have tried to get a grip on the new, hard reality, it has been suggested that the pandemic might have made people more digitally literate (Jeleński 2020).

However, the tense, difficult situation attracted many wrongdoers and criminals, who have abused the fact that so many people were bound to use the Internet for work, learning, training, communication, purchasing necessities, etc.; that they have become utterly dependent on the Internet (Fidler 2020). Along with the massive increase in the number of videoconferences, the popularity of online shopping, banking, and so on, the amount of malicious software, phishing e-mails and ransomware attacks, along with the staggering amount of COVID-19-related fake news has been disturbingly rising, with the occurrence of some types of attack increasing fivefold since the beginning of the pandemic (Rementeria 2020)(WHO 2020). Again, the people with the lowest levels of digital literacy, who already might have been experiencing some forms of exclusion, have become the most vulnerable group and their security and privacy may be compromised, by various, malicious cyberspace actors (Gerg 2020; Pawlicka et al. 2020b, a, 2021a).

### Legal background

Currently, there is still an ongoing debate (also at the United Nations level) tending towards stating that the same human rights that apply offline should also be protected online, and that access to the Internet should be considered a human right, in particular in the context of the freedom of expression covered under article 19 of the Universal Declaration of Human Rights (Article19, 2018). One of the first documents in which the access to the Internet was considered as a human right was the Report of the Special Rapporteur on the promotion and protection of the right to freedom of opinion and expression, Human Rights Council (Rue 2011).

Later in 2016, the Human Rights Council published the resolution on the promotion, protection and enjoyment of human rights on the Internet (UN General Assembly Human Rights Council, 2016). In this document, the importance of empowering all women and girls by enhancing their access to information and communications technology, promoting digital literacy and the participation of women and girls in education and training on information and communications technology, and encouraging women and girls to embark on careers in the sciences and information and communications technology was directly emphasized. The article 5 of the Resolution suggested that the states should make efforts to bridge the gaps of digital divides, including those of gender.

The problem of gender in this respect was already expressed in 2017 by the United Nations High Commissioner for Human Rights in the General Assembly Report on Promotion, protection and enjoyment of human rights on the Internet: ways to bridge the gender digital divide from a human rights perspective (UN General Assembly Human Rights Council, 2016). In the report, it was stated that lower digital skills and lower digital literacy can directly influence lower position of women in the labour market, and in leadership positions.

In 2018, the Human Rights Council approved yet another document concerning the rights to the Internet access, namely the resolution on the promotion, protection and enjoyment of human rights on the Internet at the 38^th^ Session of the Human Rights Council in Geneva. In this document, the emphasis is also put on the need to address the gender-based digital divides. Point 5 of the resolution encourages all the countries to put efforts into bridging digital divides, including the gender digital divide. The resolution also calls for enabling online environment that is safe for all, and facilitates affordable and inclusive education.

## Theoretical background

### Digital competences as a key factor

In order to construct and design the research study and tool, the concept of digital skills had to be defined.

For the sake of this study, the notions of digital skills, digital competences and digital literacy have been used interchangeably. When building the tool, the definitions by the European Parliament and the Council has been assumed. They have come up with a set of eight *Key competencies for Lifelong learning*. The competencies have been described as a mixture of knowledge, skills and attitudes that every person requires to derive personal fulfilment, develop as a person and be an active, upright citizen. They are also necessary for employment and prevent being socially excluded. The eight key competences comprise:

1. Communication in the mother tongue
2. Communication in foreign languages
3. Mathematical competence and basic competences in science and technology
4. Digital competence
5. Learning to learn
6. Social and civic competences
7. Sense of initiative and entrepreneurship
8. Cultural awareness and expression.

European Parliament and the Council have recommended the Member States provided these competencies in all their strategies of lifelong learning, as a way of preparing young people for adulthood and being a basis for further learning and working life, and updating and developing the competencies of all the adults. It has also been recommended to make adequate provision for the citizens who require particular assistance in order to realize their potential, whether it be owing to personal, cultural, social or economy-related circumstances (Eur-Lex 2006).

The digital competence is defined as involving the confident and critical use of Information Society Technology (IST) for work, leisure and communication. According to the recommendations, possessing digital competence means the individuals can understand the nature, role and opportunities of digital environment in their daily lives; both in the professional and personal contexts (Eur-Lex 2006). Digital competence is a broad and vague term; therefore, the skills it encompasses have been organized into a clear, conceptual framework—Digital Competence Framework for Citizens, called DigComp. Pursuant to the updated version of the model (known as DigComp 2.0), the skills that digital literacy comprises fall into five categories; altogether there are 21 particular skills in this model. They have been shown in Table 2.

**Table 2:** The competence areas of digital literacy and the particular skills, based on DigComp 2.0 (Carretero et al. 2017)

Competence area	Skills
1: Information and data literacy	1.1 Browsing, searching, filtering data, information and digital content
	1.2 Evaluating data, information and digital content
	1.3 Managing data, information and digital content
2: Communication and collaboration	2.1 Interacting through digital technologies
	2.2 Sharing through digital technologies
	2.3 Engaging in citizenship through digital technologies
	2.4 Collaborating through digital technologies
	2.5 Netiquette
	2.6 Managing digital identity
3: Digital content creation	3.1 Developing digital content
	3.2 Integrating and re-elaborating digital content
	3.3 Copyright and licenses
	3.4 Programming
4: Safety	4.1 Protecting devices
	4.2 Protecting personal data and privacy
	4.3 Protecting health and well-being
	4.4 Protecting the environment
5: Problem solving	5.1 Solving technical problems
	5.2 Identifying needs and technological responses
	5.3 Creatively using digital technologies
	5.4 Identifying digital competence gaps

### State-of-the-art of digital literacy studies

The literature sources present a number of studies related to digital literacy and competences in the context of universities or higher education. In their research, (Shopova 2014) scrutinised the levels of digital literacy amongst students. Although digital competences were deemed crucial for enhancing the learning process, the majority of young people who took part in the experiment lacked many of the much needed skills, such as e.g., using the Internet to solve tasks or scientific problems. The author of Noh (2017) also studied university students, in the context of their information use behaviour. In turn, Tang,Chaw (2016) examined the digital literacy of university students in the context of blended learning. The researchers concluded that digital competences are a prerequisite for successful participation in blended learning. In their work, McGuinness and Fulton (2019) discussed the digital literacy in higher education, again in the context of blended learning. Over eighty students took part in the study; the results re-stated the usefulness of digital literacy in the process of acquiring knowledge.

The only work which touched upon the digital literacy of teachers in the pandemic was the paper by Santi Susanti Rachmaniar (2020). It checked if and how the digital competence levels shifted in the teachers of an elementary school since the onset of the COVID-19 pandemic. Indeed, some of the studied people reported that they gained new digital skills whilst preparing their lessons for students. Still, a number of teachers still had difficulty in this domain and had to ask the students’ parents for assistance.

However, to date, to the authors’ best knowledge, no study of the level of digital literacy of university teachers/ educators amidst the pandemic has been conducted.

### Data mining

Data mining, sometimes referred to as KDD (knowledge discovery in databases) consists in extracting unrevealed information from substantially vast datasets. In order to extract patterns and find order in the historical data, intelligent methods are often applied (Bhargava and Selwal 2013). Association rule mining is a kind of data mining. It is utilised in order to discover association relationships between the items belonging to big datasets. The basic association rule is X $\rightarrow$ Y; it means that if X is true of an instance in a dataset, then Y is true of it, too. In this relation, X is called an antecedent and Y—the consequent. Antecedents are understood as the items that appear first and all the consequents as the ones that follow them. The level of significance of the association is measured using three indicators—support, confidence and lift. Support explains the level of popularity of a given combination of items (i.e., an itemset) within a dataset. It is the proportion of the number of occurrences of the itemset of X and Y to the total number of items in it. It is expressed by the Formula 1:

1 $$\begin{aligned} Support=\frac{freq(X,Y)}{N} \end{aligned}$$

(Agrawal et al. 1993).

Confidence is the measure of how likely the item Y is to appear together with the item X; it is expressed by the proportion of the number of instances when X and Y appear together vs. the number of times X appears. The formula of confidence is:

2 $$\begin{aligned} Confidence=\frac{freq(X,Y)}{freq(X)} \end{aligned}$$

(Agrawal et al. 1993)

As this measure may misrepresent the significance of an association (especially, if item Y is popular as well), the third measure is applied, namely the lift. Lift says how likely the item Y is to appear together with item X, taking into account the popularity of the item Y as well. Lift is expressed by the formula:

3 $$\begin{aligned} Lift=\frac{Support}{Support\,(X) \times (Support\,(Y)} \end{aligned}$$

(Gokul 2020)

In other words, lift is the measure which assesses the strength of the association (Gokul 2020). If the lift is higher than 1, it means that the occurrence of X does lead to Y. The higher the lift, the stronger the association. A lift value close to 1 shows that one item does not affect the other. Consequently, if the lift is lower than 1, then the occurrence of X has a negative effect on the occurrence of the item Y (IBM Knowledge Center, 2021). For the sake of this particular study, it has been decided apply the apriori algorithm. The algorithm is one of the most popular ones used for association rule mining. It was first introduced by Agrawal (1994) and described in detail by Bhargava and Selwal (2013). Apriori assumes that all subsets of a frequent itemset must be frequent; conversely, if an itemset is not frequent, then its supersets will be infrequent, too. It is worth noting that the thresholds/ levels of frequency are decided upon by the person using the algorithm, based on experience, experiment, expert advice, needs, etc. The apriori algorithm has been often used for the so-called market basket analysis, that is analysisng transactions in search for the items frequently bought together. It allows one to find interesting, often surprising associations within large datasets. It is also said to be user-friendly and easy to use; however, it may require a lot of resources and computation time if applied on a large dataset and with minim measures’ thresholds kept very low. The particular algorithm was chosen for this experiment in hope it would yield interesting results, help confirm or reject the research assumptions and answer the following research questions.

## Materials and methods

### Part 1: The dataset and research group

It was decided that the first study of the series will be conducted on women who are university teachers/ educators and/or scientists by profession.

This selection was made based on the fact that women score more poorly in digital literacy tests (EUROSTAT, 2019) (Jiménez-Cortés et al. 2017). Moreover, teachers (including the higher education ones) have been reported not to utilise the available digital tools which would enhance the learning outcomes (De Pablos Pons 2010). The lack of proper digital skills was pointed out as one the reasons for such a situation (García-Pérez et al. 2016). Finally, universities in Poland were made to go online by law since 12^th^ March 2020 (Ustaw 2020),(of Science and Education, 2020)

Thus, the women working at universities, known to be forced to work remotely, should show the increase in their levels of digital literacy and cybersecurity awareness.

### The research methodology and the design of the data gathering process

In order to conduct the study, a questionnaire was constructed. It consisted of three parts, the first one asking the questions about the scientific title, the scientific field the studied person worked in, their age, place of residence (a city or a country) as well as if they worked as university teachers/educators, scientists, or both.

The subsequent section concerned the fact if they worked remotely, and if they did not, the survey finished. Then, there were the questions on the perceived (self-reported) level of the digital skills of the studied individual; i.e., the question about the perceived general level, the five components of digital literacy according to the DigiComp 2.0 Framework and if the employer helped/supported them in gaining the necessary skills. The section finished with the question if the person felt their perceived level of digital literacy increased during the COVID-19 pandemic.

The last section of the questionnaire touched upon the aspects of cybersecurity. The first question aimed at checking whether the studied person felt safe when using the cyberspace and its tools. Then, it was checked whether the person’s employer had made them aware of the possible threats to their assets and privacy, that may come with their remote work. Finally, in the last question, the individual was to assess if their perceived level of cybersecurity awareness increased during the COVID-19 pandemic.

The primary version of the questionnaire was an online tool. It was distributed amongst the people belonging to the target group by various means: using instant messages, Facebook groups, or asking the individuals personally and then sending them the link to the questionnaire via e-mail. Each person was informed of the fact that the study was anonymous, GDPR compliant and no personal data was collected/saved (Pawlicka et al. 2020b). This broad information gathering campaign took place in October 2020.

### The dataset at a glance

Altogether, 380 women responded to the questionnaire. To the authors’ knowledge, this has been the first scientific study of the digital skills and cybersecurity awareness of women academics and scientist in the times of the pandemic, and one of the largest studies of digital competences and cybersecurity awareness of women academics in general.

#### Subjects from whom data was collected

**Scientific title** The majority of respondents hold the doctoral degree (63%). 18% of them has the PhD/ScD hab. (“habilitation”—a title used in some countries with seniority between a doctor and Full Professor) title; 17%—MA; 2% are full Professors, and 1% of them hold other titles.

**Scientific field** The majority of the studied women deal with social sciences (41.6%). Almost a quarter of the respondents (23.5%) work in exact or natural sciences. The third largest group belongs to humanities $-$19.8%. The remaining fields were: engineering or technical studies—8.7%, agriculture—4.1%, art 2% and theological studies—0.3%.

**Age of the respondents** The studied women were aged from 25 to 70 years old. The average age was 38.8 years old; the median age was 38.

**Place of residence** The vast majority of respondents live in the city (89%).

#### Digital competence

**General level** Most respondents assess their perceived level of digital competence in a positive way, as they chose either “very high”(27%) or “high” (56%). Only 1% of the respondents believe their overall level of digital competence is low. It is worth noting that no respondent found the level of their digital literacy to be very low. The rest of the studied women (16%) believe they have average digital competence.

Then, the level of the particular components of digital competence (according to DigiComp 2.0) was measured. Each of the respondents was given the description of the component, along with the examples of practicing it. They were supposed to assess the level of competence within a particular component using the grade from 1 to 5, where 5 meant“very high”, 4 - “high”, 3 -“it is hard to tell”, 2 -“low”, and 1 - “very low”.

**The assessment of the digital literacy components**

**Fig. 1:**
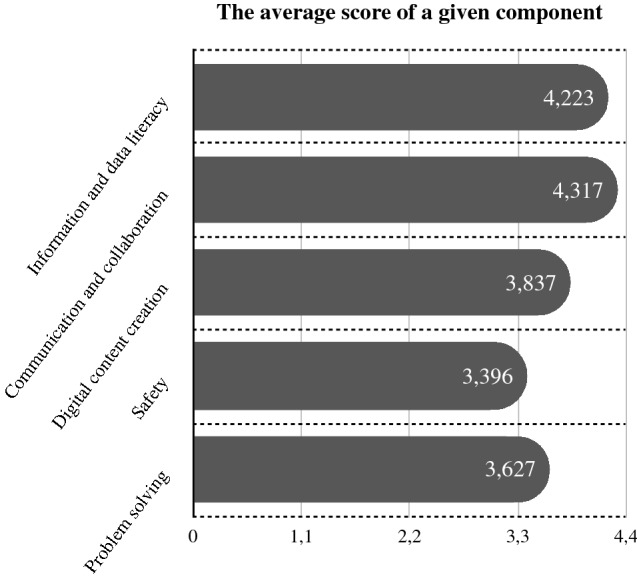
The comparison of the average score received by each of the digital literacy components

Figure 1 shows the imbalance in respondents’ digital skills; there are distinct differences between the perceived levels of particular components of digital literacy. Communication and collaboration skills scored the highest, whilst the average score for safety was almost a point lower.

**The employers’ support** The next question concerned the fact if the respondents’ employers have supported them in their transitioning to working remotely using the Internet; by providing various forms of training, educational materials, tutorials or by providing access to resources/helpdesks. Almost three quarters of employers (73%) have done it.

**The pandemic and the increase in the perceived level of one’s digital skill** When asked if their perceived, overall level of digital competence had increased during the COVID-19 pandemic, almost two thirds of the respondents (61%) answered “definitely yes” or “rather yes”. Only about a quarter (26%) of the studied group believe their digital skill levels have not increased (“rather not” - 22% and “definitely not” - 4%). The rest is not able to assess if their skills have increased or not.

#### The level of cybersecurity awareness

The following part of the study concerned the aspects of cybersecurity.

**Feeling safe when working online** Most respondents (57%; “definitely yes” - 6%, “rather yes” - 51%) feel that both them and their property are safe when they use the Internet for working online. About a quarter of them cannot decide whether they feel safe or not. The remaining 17% do not feel safe when working online (14% - “rather not” and 3% - “definitely not”).

**The employers’ role in raising the cybersecurity awareness level** When asked if their employer had made them aware of any cybersecurity measures or possible cyber threats, most respondents (64%) denied.

The last question concerned the fact if the respondents felt their level of cyberse- curity awareness had increased during the COVID-19 pandemic. The greatest group believes it has not raised (43%, “rather not” - 35%, “definitely not” - 8%). About a quarter of the respondents (26%) find the awareness level to have increased (“rather yes” - 22%, “definitely yes” - 4%). Almost one third of the studied women (32%) cannot decide whether cybersecurity awareness level has shifted, or not.

#### Part II: The data mining process

As evident from the data gathered, most respondents (over 60%) believe their digital skill level has raised during the pandemic. One may believe that this shift is caused by people having to deal with the digital issues by themselves, as suggested in Jeleński (2020). However, in almost 75% cases, people admitted to having been supported by their employers in transitioning to working online, too. At the same time, only about a quarter of them believe their cybersecurity awareness level has increased when working remotely via the Internet, and most respondents claimed that their employers had not made them aware enough of the cybersecurity matters. In order to find out whether there actually is any relation between these factors, the data was processed with the association rule mining algorithm.

**Experimental setup and preparing the dataset** In order for the dataset to be ready for applying the algorithm, all the data items were changed to categorical ones. The values for minimum support, confidence and lift were selected in an experimental way, in order to reflect the associations in the most accurate way possible and economise on the computational time. The final values were: minimum support = 0.025, minimum confidence = 0.3, minimum lift = 6.

**The results** After the data was processed by the apriori algorithm, a list of 11 association rules was created. Then, it was sorted according to the lift value, i.e., the higher the value, the stronger the association, according to the algorithm. The results have been presented in Table 3.

**Table 3:** The association rules found in the course of the experiment; min. support = 0.025, min. confidence = 0.3, min. lift = 6

No.	Rule number	The rule	Support	Confidence	Lift
1	4	If a person feels “definitely safe” then they “definitely” had considered their online safety, “definitely” try to protect themselves online and had never fallen victim to cyberattacks.	0.028871391	0.47826087	8.677018634
2	2	If a person feels “definitely safe” then they “definitely” try to protect themselves online and their employer had made them aware of the cybersafety issues.	0.028871391	0.47826087	7.592391304
3	9	If a person feels “definitely safe” then they “definitely” try to protect themselves online, their employer had provided them with support and/or training of their digital skills and their employer had made them aware of the cybersafety issues.	0.026246719	0.434782609	7.202268431
4	1	If a person is 26–29 years old, then they are a MA and assess their digital skill level in a very positive way.	0.028871391	0.35483871	7.115449915
5	8	If a person is 26–29 years old, then they live in a city, are a MA and assess their digital skill level in a very positive way	0.028871391	0.35483871	7.115449915
6	6	If a person is 26–29 years old, then they live in a city, are a MA and had never fallen victim to a cyberattack	0.028871391	0.35483871	6.759677419
7	7	If a person feels “definitely safe” then they assess their digital skill level in a very positive way, “definitely” try to protect themselves online and their employer had provided them with support and/or training of their digital skills	0.026246719	0.434782609	6.626086957
8	0	If a person is 26–29 years old, then they are a MA and their digital skill level had “rather” raised during the pandemic	0.026246719	0.322580645	6.468590832
9	3	If a person is 26–29 years old, then they live in a city, their digital skill level had “rather” raised during the pandemic and are a MA.	0.026246719	0.322580645	6.468590832
10	10	If a person feels “definitely safe”, then they then they “definitely” had considered their online safety, they live in a city, “definitely” try to protect themselves online, their employer had provided them with support and/or training of their digital skills	0.026246719	0.434782609	6.1352657
11	5	If a person feels “definitely safe”, then they then they “definitely” had considered their online safety, “definitely” try to protect themselves online, their employer had provided them with support and/or training of their digital skills	0.028871391	0.47826087	6.073913043

## Discussion of the results

The COVID-19 pandemic has influenced almost every aspect of people’s lives. Many routines and activities have moved to the online domain and may remain this way for a longer time, maybe even forever. This shift has challenged people’s digital literacy and made them think about their online assets being secure. The conducted study aimed at checking if both the aspects improved, as a surprising side effect of the life-threatening global pandemic, and which factors are associated with the self-reported digital skill level and cybersecurity awareness level/feeling safe when working online.

### The answer to the research question 1

As anticipated, the perceived level of people’s general digital literacy has raised during the COVID-19 pandemic; over 60% of the respondents believed so. The association rule mining study, however, did not find a significant association between having to work remotely and the actual increase in the level; rather, it was related to the employers’ support and workplace trainings.

### The answer to the research question 2

Despite being forced to utilize online services in almost every aspects of their lives and becoming almost totally dependent on the Internet, people do not seem to have become more aware of the possible cyberthreats and the cybersecurity measures aimed at preventing them. This should be worrisome, as the study of the particular components of digital literacy had shown safety is the aspect which people know the least about. There is a concern to be had, as at the same time, they feel relatively safe, which might lead to them not being alert enough and falling victim to various types of cyber exploits. Only about a quarter of the respondents believe their cybersecurity awareness level has increased when working remotely via the Internet; this may be related to the fact that, as it turns out of the study, most employers seem not to put enough emphasis on the cybersecurity-related matters. A similar conclusion was reached as a result of the association mining study—the respondents who were made aware of the cybersecurity matters by their employers, reported to be feeling safe and secure when working online.

This is the clear hint for the employers that they need to include the cybersecurity awareness-enhancing frameworks in their training/workplace education agenda. In the long run, this will help people protect their data and property when working online.

### The answer to the Research question 3

As expected, the youngest respondents were the ones who reported to be having the highest level of digital skills; this goes in accordance with the previous findings. The algorithm also found a strong the association between the age and being a MA; this came as no surprise, as the respondents aged 26–29 are usually in the process of gaining their PhDs and further titles.

### The answer to the research question 4

The level of one’s perceived security when working online is strongly associated with the fact if their employer provides digital skills and cybersecurity-related training and support, if they consider their safety and make active efforts in order to protect themselves.

### Threats to validity and the limitations of the study

As for construct validity, it is believed that the language of the questionnaire and questions were well understood. This aspect was evaluated by asking 5–10 first subjects (who were known to authors) to answer the survey. Regarding external validity, the study was conducted amongst women who are educated (as academics) and mostly dwell in cities; the authors are aware that the study did not encompass the representatives for lower classes/education level. In order to obtain the fullest picture of the matter, the study would need to include the people belonging to the lowest class, living in the country, as well as female primary school teachers. The authors have already planned to conduct such a study in the upcoming time and employ other data mining algorithms, and will share the updated results afterwards.

## Conclusions

### Practical and theoretical implications

There is no doubt that the pandemic-related crisis, which has also resulted in an economic crisis and increased unemployment, has revealed digital and technical skills deficits. In the case of this work, they were uncovered among women working in such an important area as science. Women employed in this sector need to acquire digital competences if they want to retain their job posts, but also if they wish to teach today’s young people. Generation Z, iGen, iGeneration, generation XD—these are the terms used to describe the digital generation now entering adulthood, who do not know a world without the Internet and prefer to spend their time on the phone rather than amongst other people. This generation is composed of the people born after 1995 (the year the Internet was commercialised), or 2000. They are the first generation to have permanent access to the Internet.

In complementing and upgrading their digital competences, some of the female scientists surveyed are able to cope on their own, but others need immediate help in the form of action from employers (universities, colleges). This issue implies further analysis on the educational role of organisations.

Complementing qualifications and skills in this area is also necessary due to the phenomenon of technological exclusion, which increases dramatically during the pandemic period. The women surveyed need to intensify their professional development in this area in order to equalise opportunities. There is no other way to keep a job.

The article also highlights aspects related to digital security The insufficient level of competence in dealing with digital threats points to the need for awareness and education, especially since the scale of threats from the digital world is constantly increasing. Not everyone is able to increase their competences quickly enough through individual self-education, which is necessary to deal effectively with multi-faceted e-risks.

Education in digital competences, including education in digital safety, is an action which should be taken as soon as possible in the Polish higher education environment in order to mitigate the negative effects of the crisis, but also to use this moment to equalise the chances of women on the labour market in the digital economy of the future.

SPARTA, a Horizon 2020 cybersecurity pilot project, funded by the European Commission, is an example of such a desired initiative, as a significant effort is placed in it to help tackle both of the challenges presented in this study. A range of actions undertaken and systems are being put in place to help mitigate the “gender gap”. This starts with embedding actions inside the project itself, and then communicating the principles and values outside the project to help specifically address the female public, in both dissemination and communication activities. The female participation in all training related activities during the project is prioritized, so as to focus on and incentive female participation, involvement and uptake. Female mentorships programs within SPARTA partner cybersecurity research teams are being created. The project also strives to understand and correct social barriers related to female participation in all levels of the cybersecurity workforce. The results of this investigation illustrate the immense importance of innovative initiatives like the “Women in Cyber Campaign” implemented in SPARTA (Lindner et al. 2020).

### Final remarks

This particular study concerned women-scientists and/or university teachers, thousands of whom had been forced to start working online when the pandemic broke out. Most of the respondents report that their digital literacy has increased. As women used to score more poorly in the digital skills tests before the pandemic, it may turn out that the global crisis contributed to their gaining more competence. It might even turn out that this will be one of the very few benefits of this terrifying situation. However, the shift did not happen by itself. The association rule mining study has shed some light on the significance of the workplace training and support; there is a strong relation between the employees feeling their competence rises and the fact if their employers provided them with the opportunity to gain the skills.

Moreover, people’s cybersecurity/safety skills scoring the lowest, along with the lack of emphasis on the cybersecurity matters at workplace are alarming. Cybersecurity skills need to be addressed more, as, the lack thereof may lead to disastrous results, like personal harm or financial losses, even after the pandemic finishes.

Finally, cybersecurity awareness is not something which appeared alongside the pandemic-related spike in the amounts of cyber-mischief. Apart from the support at one’s workplace, a person does need to make active efforts in minding and protecting their cybersecurity level in order to feel as safe and confident as possible when working in a remote manner.

## Data Availability

The datasets generated during and/or analysed during the current study are available in the Harvard Dataverse repository, 10.7910/DVN/B2PZDH; they are also available from the corresponding author on reasonable request.
